# Draft Genome Sequence of Marinobacter hydrocarbonoclasticus NCT7M, Obtained from the Microbiome of a Marine Copepod

**DOI:** 10.1128/MRA.01459-19

**Published:** 2020-03-12

**Authors:** Ryan A. Nuttall, Gaurav Sharma, Pia H. Moisander

**Affiliations:** a Department of Biology, University of Massachusetts Dartmouth, North Dartmouth, Massachusetts, USA; b Institute of Bioinformatics and Applied Biotechnology, Electronic City, Bengaluru, Karnataka, India; University of Southern California

## Abstract

The gammaproteobacterium Marinobacter hydrocarbonoclasticus NCT7M was cultivated from the copepod Acartia tonsa, collected from the coastal western North Atlantic Ocean. The genome was assembled into 45 contigs for a total of 4,128,590 bp, a GC content of 57.3%, and 3,890 protein-coding genes. The genome contains the full gene cluster for denitrification.

## ANNOUNCEMENT

Culture isolation targeted bacteria potentially capable of dissimilatory nitrate reduction in association with marine copepods (1). Cultures were established from the copepod Acartia tonsa, collected from the surface waters of the temperate western North Atlantic Ocean in January 2016 (41.57N, −70.80W). Sequencing reported here identified strain NCT7M as Marinobacter hydrocarbonoclasticus (kingdom, *Bacteria*; phylum, *Proteobacteria*; class, *Gammaproteobacteria*; order, *Alteromonadales*; family, *Alteromonadaceae*). To establish cultures, individual copepods were spread on nitrate-chitin plates and incubated anaerobically in the dark at 25°C. A liquid enrichment culture was initiated from a streaked colony and then grown aerobically at 25°C with shaking in modified marine broth (sea salts [catalog number S9883, Sigma-Aldrich, St. Louis, MO], 64 g liter^−1^; peptone, 8 g liter^−1^; K_2_HPO_4_, [100 μM]; NaNO_3_^−^, [200 μM]; NH_4_Cl, [200 μM]). Genomic DNA was extracted using the DNeasy plant minikit (Qiagen, Valencia, CA, USA). A genomic library was prepared using the Nextera XT DNA library preparation and index kits (Illumina, San Diego, CA) and sequenced on the Illumina MiSeq 2 × 250-bp paired-end platform at Tufts University (Boston, MA). Default parameters were used for all software unless otherwise specified. Adaptor sequences were trimmed from both ends of the resulting 889,443 reads. The reads were trimmed to remove positions with a Phred score of <28 using Trimmomatic v0.35 (2). These filtered reads (564,510 reads; 63.5% of total) were assembled with SPAdes v3.9.1 (3). The contigs were cross-checked for contamination with the Kraken-based taxonomic sequence classification system (v1.0) (4), and only contigs matching *Marinobacter* spp. were kept in the assembly (53% of the entire contig length, constituting 2.54% of all contigs). The final draft genome consists of 4,128,590 bp assembled in 45 contigs with a GC content of 57.3% and 13.0× coverage. The final assembly was run with CheckM (5), which showed maximum closeness with c__Gammaproteobacteria (UID4444) with 99.78% completeness and only 0.42% contamination. The contig sizes range from 594 bp to 554,269 bp, with an *N*_50_ value of 223,815 bp for the assembly. RAST annotation v2.0 (6; http://rast.theseed.org/FIG/rast.cgi) identified 3,890 protein coding sequences, 45 tRNA genes, and 5 rRNA genes (3 5S, 1 16S, and 1 23S rRNA). Of the 3,890 protein-encoding sequences, 2,671 have a nonhypothetical function, and 1,219 are linked to subsystems. The subsystems found, including virulence and defense, iron acquisition, motility and chemotaxis, secondary metabolism, nitrogen and phosphorus metabolism, and stress response, potentially increase fitness and competitiveness in animal associations.

The closest 16S rRNA gene match to NCT7M was *M. hydrocarbonoclasticus* SP17 (ATCC 49840) (99.93%; NCBI BLASTn online), and matches to other *M. hydrocarbonoclasticus* strains were highly similar. Digital DNA-DNA hybridization (DDH) (7; http://ggdc.dsmz.de/ggdc.php) similarity values ranged from 73.2% to 71.8% between NCT7M and other *M. hydrocarbonoclasticus* genomes and from 18.8% to 20.5% with other *Marinobacter* spp. Average nucleotide identity (ANI) (8; http://enve-omics.ce.gatech.edu/ani/) values of NCT7M and other *M. hydrocarbonoclasticus* genomes ranged from 96.7% to 97.1% and from 77.8% to 82.4%, respectively, with other *Marinobacter* spp. (Table 1). *Marinobacter* spp. are Gram-negative, rod-shaped, moderately halophilic bacteria that often have the capacity to use hydrocarbons for energy generation and carbon acquisition (9).

**TABLE 1:** Comparison of *M. hydrocarbonoclasticus* NCT7M to *Marinobacter* spp. using ANI and DDH^*a*^

Organism and strain	ANI (%)	DDH (%)	GenBank accession no.	Origin
*M. hydrocarbonoclasticus* SP17 (ATCC 49840)	97.1	73.2	GCA_000284615.1	Polluted sediment
*M. hydrocarbonoclasticus* 114E	96.9	72.3	GCA_003337655.1	Wastewater facility
*M. hydrocarbonoclasticus* 114E_o	96.9	72.3	GCA_003315555.1	Wastewater facility
*M. hydrocarbonoclasticus* 105B	96.7	71.8	GCA_003337515.1	Wastewater facility
*M. hydrocarbonoclasticus* DSM 50418	96.9	71.8	GCA_003634635.1	Seawater
*M. psychrophilus* 20041	77.8	18.8	GCA_001043175.1	Sea ice
*M. salarius* R9SW1	80.3	20.4	GCA_000831005.1	Seawater
*M. salinus* Hb8	79.1	19.1	GCA_001854125.1	Tidal flat sediment
*M. excellens* LAMA 842	82.4	22.0	GCA_001574445.1	Marine sediment
*M. nanhaiticus* D15-8W	79.0	20.5	GCA_000364845.1	Marine sediment

a DDH was calculated using formula 2 (15).

AntiSMASH 5.0 (10; http://antismash.secondarymetabolites.org) identified pathways for ectoine, an unidentified siderophore, and two beta-lactones. NCT7M has gene clusters for denitrification (11), which are identical with those found in four other *M. hydrocarbonoclasticus* genomes (strains 114E, 114E_o, 105B, and DSM 50418), suggesting conservation of the pathway across the species. Several *M. hydrocarbonoclasticus* strains can denitrify when oxygen is present (12–14); thus, NCT7M may also denitrify aerobically. NCT7M appears to be the first *M. hydrocarbonoclasticus* strain cultivated from copepod association. The genome should contribute to investigations of bacterium–animal associations and to understanding of the importance of aerobic denitrification in the marine environment.

### Data availability.

This whole-genome shotgun project has been deposited at DDBJ/ENA/GenBank under the accession number WBMP00000000. The version described in this paper is version WBMP01000000. The data are available under BioProject number PRJNA573915 and BioSample number SAMN12830880. The reads are available under number SRR10424827.
